# Water Resources Carrying Capacity Evaluation and Diagnosis Based on Set Pair Analysis and Improved the Entropy Weight Method

**DOI:** 10.3390/e20050359

**Published:** 2018-05-11

**Authors:** Yi Cui, Ping Feng, Juliang Jin, Li Liu

**Affiliations:** 1 Stage Key Laboratory of Hydraulic Engineering Simulation and Safety, Tianjin University, Tianjin 300072, China; 2 School of Civil Engineering, Hefei University of Technology, Hefei 230009, China

**Keywords:** water resources carrying capacity, evaluation and diagnosis, vulnerability index, information entropy, set pair analysis, set pair potential, index weight

## Abstract

To quantitatively evaluate and diagnose the carrying capacity of regional water resources under uncertain conditions, an index system and corresponding grade criteria were constructed from the perspective of carrying subsystem. Meanwhile, an improved entropy weight method was used to determine the objective weight of the index. Then, an evaluation model was built by applying set pair analysis, and a set pair potential based on subtraction was proposed to identify the carrying vulnerability factors. Finally, an empirical study was carried out in Anhui Province. The results showed that the consistency among objective weights of each index was considered, and the uncertainty between the index and grade criterion was reasonably dealt with. Furthermore, although the carrying situation in Anhui was severe, the development tended to be improved. The status in Southern Anhui was superior to that in the middle area, and that in the northern part was relatively grim. In addition, for Northern Anhui, the fewer water resources chiefly caused its long-term overloaded status. The improvement of capacity in the middle area was mainly hindered by its deficient ecological water consumption and limited water-saving irrigation area. Moreover, the long-term loadable condition in the southern part was due largely to its relatively abundant water resources and small population size. This evaluation and diagnosis method can be widely applied to carrying issues in other resources and environment fields.

## 1. Introduction

Water resources are not only basic natural resources, but also strategic economic resources and ecological control factors. The factors have become vital controlling elements in limiting the development of the global economy and society in the 21st century [1]. Meanwhile, with economic development and population growth, the contradiction between the rapid development of the economy, the steady health of the ecology and the sustainable utilization of the water resources has become increasingly prominent [2]. The carrying capacity of regional water resources is a comprehensive reflection of interactions between water resources, social economy, and ecological environmental systems, which are crucial indicators for evaluating regional water resource security [3]. When the carrying capacity of water resources exceeds a certain threshold, it will seriously restrict sustainable development of the economic society and, meanwhile, pose a direct impact on food and ecological security [1]. Therefore, the accurate evaluation and diagnosis of the regional water resources carrying capacity are significant for guiding the rational utilization of water resources and guaranteeing the water security in a context of economic and social development.

The concept of the carrying capacity originated from the field of ecology, where it is used to quantitatively describe the maximum number of an individual species that can be maintained in a special circumstance region [4]. In the early 1980s, the meaning of resource carrying capacity was put forward by United Nations Educational, Scientific and Cultural Organization (UNESCO) due to the growing contradiction between the economic development and resource shortages. The notion of the water resources carrying capacity was first proposed by the Xinjiang Water Resources Soft Science Project Team in 1989 [1]. Subsequently, the connotation of the water resources carrying capacity was further discussed. Some scholars thought that the water resources carrying capacity indicates that water resources are needed to maintain the coordinated development of the social-economic system [5,6]. Several others considered that the water resources carrying capacity is the maximum threshold of water resources to sustain human activities [7,8]. Actually, the water resources carrying capacity is a concept with twin attributes of nature and society, but is not limited to these [9]. Based on these and previous research, the water resources carrying capacity is regarded as the maximum sustainable socioeconomic scale that can be supported by water resources while maintaining defined environmental conditions in this study.

The research on the quantitative evaluation and diagnosis of water resources carrying capacity has been widely concerned, and it is always a key and challenging issue for the sustainable utilization of water resources [2,10]. To evaluate and diagnose the carrying capacity of regional water resources is to build an index system, grade criterion, and evaluation model from the aspect of the carrying subsystem based on the comprehensive analysis of carrying elements, and then to judge whether the situation of regional water resources could support the sustainable development of the social economy and, meanwhile, maintain a good condition of the ecological environment, as well as identifying the main factors that cause the overload of water resources. It is a vital part of constructing carrying capacity monitoring and early warning mechanisms of regional water resources, providing the foundation for realizing the strictest water resources management [11,12,13]. Meanwhile, it contributes to proposing reasonable suggestions for guaranteeing the coordinated development among the population, economy, and environment in a study area [1,7].

Currently, the methods for quantitatively evaluating the regional water resources carrying capacity have been proposed and improved, which mainly include a comprehensive index method [14], a fuzzy comprehensive evaluation method [15], a principal component analysis method [16], a projection pursuit method [17], a driving force-pressure-state-influence-response-management concept model [18], and a matter element extension model [19]. However, as a typical complex system, water resources carrying capacity is influenced by many factors from various aspects, such as water resource conditions, social economy, the ecological environment, and so on [20]. Therefore, currently, the studies on the evaluation and diagnosis of regional water resources carrying capacity are incomplete, lacking an effective theory framework and quantitative calculation method, which is reflected by four problems, as follows: (a) There is a lack of systematic and scientific index system and corresponding grade criteria for evaluating the carrying capacity of regional water resources [10,21]; (b) The weight of the evaluation index could not be reasonably determined. In recent years, information entropy theory has been used to calculate the objective weight of the index [22,23,24]. However, the proportion of each index in the original evaluation issue is often changed with the application of this method. For example, the calculated index weights tend to be homogenized and, meanwhile, the weight of each index is determined independently, which does not consider the consistency of each index weight when the formula of extreme value normalization is applied [25,26]; (c) There is a shortage of quantitative evaluation models with strong applicability. The complex characteristics of water resources–social economy–ecological environment compound systems have not been considered completely. In addition, there are limitations in dealing with the uncertainty between evaluation index and grade criteria when using these current methods [27,28]; (d) The vulnerability index for water resources carrying is challenging to accurately diagnose. It is crucial to identify the carrying vulnerability factors and analyze the causes of the variation in carrying status for regional water resources management, but there are few quantitative studies in this field.

At present, the amount of water resources per capita in China is only 2070 m^3^/person, which is far below the average level of 7350 m^3^/person in the world [29]; the carrying situation of water resources in China is extremely grim [2,20]. As an important administrative region with rapid economic development in Eastern China, Anhui Province is located at a typical transition zone between the northern and southern climates, and it passes through three main basins: the Yangtze River Basin, the Huai River Basin, and the Xin’an River Basin. However, due to the impacts of variations in inter-annual and annual rainfall, its temporal and spatial distribution of water resources is seriously uneven [30]. The total amount of water resources in the north are obviously less than that in the south, and the per capita amount in each city differs significantly. Uneven distribution of water resources, poor water quality, and other prominent water resources problems in Anhui Province have severely restricted its sustainable economic and social development [31]. Therefore, it is vital to scientifically assess its carrying status of water resources and accurately diagnose the factors influencing its temporal and spatial variances in carrying situation. In this study, an index system and corresponding grade criteria for evaluating the carrying capacity of regional water resources are constructed from the perspective of carrying subsystem; a method that combines the information entropy theory and an improved fuzzy analytic hierarchy process is used to calculate the objective weight of the index; then, a quantitative evaluation model is established based on set pair analysis, and an improved set pair potential is proposed to identify the carrying vulnerability factor. Furthermore, empirical research is carried out in Anhui Province. This study is expected to provide guidance for regulating the carrying capacity of regional water resources.

## 2. Regional Water Resources Carrying Capacity Evaluation and Diagnosis Method

**Figure 1:**
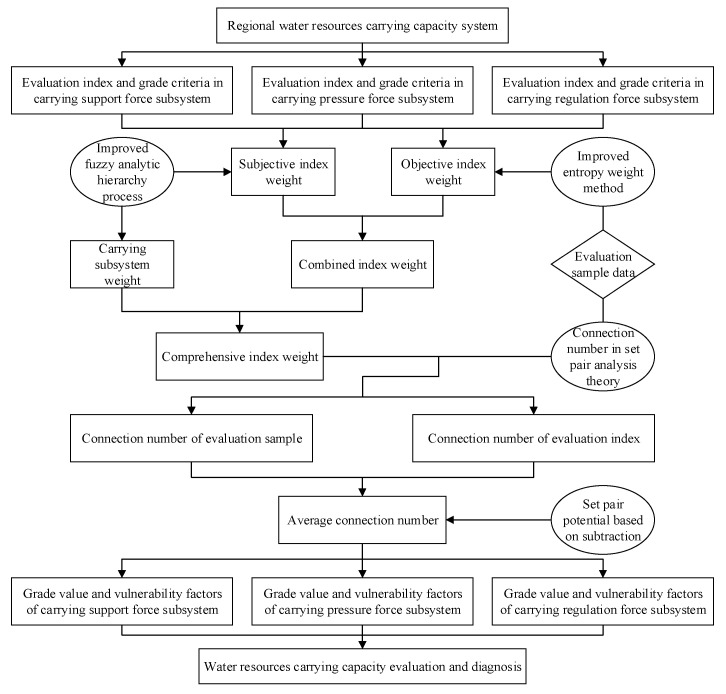
Process of regional water resources carrying capacity evaluation and diagnosis.

The process to establish the evaluation and diagnosis model included the following eight steps (Figure 1):

Step 1: According to the concept and target of water resources carrying capacity, and the principles of systematicness, representativeness, and operability [2], the evaluation index system was divided into carrying support force, carrying pressure force, and carrying regulation force subsystems from the perspective of carrying process. Based on the theoretical analysis, practical survey, literature statistics, and expert advice, these three subsystems were further decomposed into several evaluation indices, and each index was designed to own a relative ratio to be applied to different regions. The index system could be described as {*x*(*k*, *j*)|*k* = 1, 2, 3; *j* = 1, 2, …, *n_k_*}, where *x*(*k*, *j*) was the evaluation index *j* in the *k*-th subsystem; *n_k_* was the number of index in the *k*-th subsystem; *k* = 1, 2, 3 represented support force, pressure force, and regulation force subsystems; and *n* was the total number of index and *n* = *n*_1_ + *n*_2_ + *n*_3_*.* Therefore, the evaluation index samples were denoted as {*x*(*k*, *z*, *j*)|*k* = 1, 2, 3; *z* = 1, 2, …, *N*; *j* = 1, 2, …, *n_k_*}, where *N* was the number of evaluation areas.
Step 2: Based on the actual meaning, statistical characteristic, and comprehensive effect on the regional water resources–social economy–ecological environment compound system of each index, the evaluation grade criteria {*s*(*p*, *j*)|*p* = 1, 2, …, *r*; *j* = 1, 2, …, *n*} was built, where *r* was the number of grades. Without loss of generality, the level of each index was divided into three grades (i.e., *r* = 3), and *r* = 1, 2, 3 representing three types of water resources carrying status, where *r* = 1 corresponded to the loadable status representing that the regional water resources still owned enough carrying capacity, and the condition of water supply was relatively good; *r* = 2 corresponded to the critical status representing that although the degree of water resources utilization had reached a high level, there was still a certain development potential, water supply could meet the demand of social economy to some extent; and *r* = 3 corresponded to the overloaded status representing that the carrying capacity had been close to a saturated value, the potential for further development was small; moreover, if this status lasted for a long time, water shortage would occur and appropriate control measures should be taken in time.
Step 3: An improved fuzzy analytic hierarchy process based on the accelerating genetic algorithm (AGA-FAHP) [32] was used to calculate the subjective weight {*w*_s_(*k*, *j*)|*k* = 1, 2, 3; *j* = 1, 2, …, *n_k_*} and objective weight {*w*_o_(*k*, *j*)|*k* = 1, 2, 3; *j* = 1, 2, …, *n_k_*} of each index.
  - Subjective weight. The experts were invited to compare the importance of index in the *k*-th subsystem in pairs, and then the fuzzy complementary judgment matrix *A*_s_*^k^* = (*a^k^_ij_*)*_n__k_*_×*n*_*_k_* was obtained. The matrix met the criteria that 0 ≤ *a^k^_ij_* ≤ 1 and *a^k^_ij_* + *a^k^_ji_* = 1, where *a^k^_ij_* = 0.5 represented that the index *i* was as important as the index *j*, *a^k^_ij_* > 0.5 and the larger the value of *a^k^_ij_* was, the more important the index *i* was, and vice versa. The AGA-FAHP was used to test and correct the consistency of *A*_s_*^k^* and then calculate *w*_s_ (*k*, *j*). If *A*_s_*^k^* satisfied the additive transitivity, *a^k^_ij_* would meet the following formula [33]:
    (1) $$\left(a_{il}^{k}-0.5)+(a_{lj}^{k}-0.5)=(a_{ij}^{k}-0.5),\;(k=1,2,3;i,l,j=1,2,\ldots ,n_{k}\right)$$
    where (*a^k^_ij_*−0.5) can be denoted as the degree of which the index *i* is more important than the index *j*; Equation (1) indicates that this degree can be transmitted. If *A*_s_*^k^* satisfied the full consistency, the following equality would be established [33]:
    (2) $$\sum_{i=1}^{n_{k}}\sum_{j=1}^{n_{k}}|0.5(n_{k}-1)[w_{s}(k,i)-w_{s}(k,j)]+0.5-a_{ij}^{k}|/n_{k}^{2}=0$$
    where the left item in Equation (2) is the consistency index of *A*_s_*^k^*. If the result of this index was less than a critical value, it showed that *A*_s_*^k^* owned a satisfactory consistency; otherwise *A*_s_*^k^* should be corrected. Corrected *A*_s_*^k^* was denoted as *B*_s_*^k^* = (*b^k^_ij_*)*_n__k_*_×*n*_*_k_,* and the ordering weights of element in *B*_s_*^k^* were still recorded as {*w*_s_(*k*, *j*)|*k*=1, 2, 3; *j*=1, 2, …, *n_k_*}. In addition, *B*_s_*^k^* met the following formula:
    (3) $$\begin{array}{l} \min CIC(n_{k})=\sum_{i=1}^{n_{k}}\sum_{j=1}^{n_{k}}|b_{ij}^{k}-a_{ij}^{k}|/n_{k}^{2}+\sum_{i=1}^{n_{k}}\sum_{j=1}^{n_{k}}\left|0.5(n_{k}-1)[w_{s}(k,i)-w_{s}(k,j)]+0.5-b_{ij}^{k}\right|/n_{k}^{2}\\ s.t.\left\{ \begin{array}{l} b_{ii}^{k}=0.5,\;k=1,2,3;i=1,2,\ldots ,n_{k}\\ 1-b_{ji}^{k}=b_{ij}^{k}\in [a_{ij}^{k}-d,a_{ij}^{k}+d]\cap [0,1],\;k=1,2,3;i=1,2,\ldots ,n_{k};j=i+1,i+2,\ldots ,n_{k}\\ \sum_{j=1}^{n_{k}}w_{s}(k,j)=1.0,w_{s}(k,j)\in [0,1],\;k=1,2,3;j=1,2,\ldots ,n_{k} \end{array}\right. \end{array}$$
    where *B*_s_*^k^* is regarded as the optimal fuzzy consistency judgment matrix of *A*_s_*^k^* when the result of *CIC* reached a minimum value; *CIC*(*n_k_*) is the consistency index coefficient; *d* is a non-negative parameter and selected from 0 to 0.5 for guaranteeing the importance relationship between two indices [32].
    The ordering weights {*w*_s_(*k*, *j*)|*k* = 1, 2, 3; *j* = 1, 2, …, *n_k_*} and all upper-triangular elements in *B^k^* were optimization variables, and there were *n_k_*(*n_k_* + 1)/2 variables for *A*_s_*^k^* with *n_k_* order in the *k*-th subsystem. Obviously, the smaller the value of *CIC*(*n_k_*) was, the higher the consistency of *A*_s_*^k^* was. The accelerating genetic algorithm (AGA) is a global optimization method, and effective for the above optimization issue. When the result of *CIC*(*n_k_*) was less than a critical value, it indicated that *A*_s_*^k^* owned a satisfactory consistency and the calculated weights were acceptable; otherwise the parameter *d* was adjusted until *A*_s_*^k^* met a satisfactory consistency. Based on a large number of numerical experiments and relevant research [32,33], the matrix was considered to have a satisfactory consistency when the value of *CIC* was less than 0.20 in this study.
  - Objective weight. Without loss of generality, the *j*-th index in the *k*-th subsystem for the region *z*, that was *x*(*k, z, j*), was set as a non-negative value. It could be converted to a probability variable *p*(*k*, *z*, *j*) according to the definition in information entropy theory by the following formula [34]:
    (4) $$p(k,z,j)=x(k,z,j)/\sum_{z=1}^{N}\left(k,z,j\right)$$
    Equation (4) remained the proportion of *x*(*k*, *z*, *j*), which constituted the distribution of *p*(*k*, *z*, *j*).
    If the variation degree of sample series of the index *j*_1_ {*x*(*k*, *z*, *j*_1_)|*k* = 1, 2, 3; *z* = 1, 2, …, *N*} was greater than that of the index *j*_2_ {*x*(*k*, *z*, *j*_2_)|*k* = 1, 2, 3; *z* = 1, 2, …, *N*}, it indicated that the evaluation information transmitted by the index *j*_1_ was larger than that of the index *j*_2_. Therefore, the information entropy of each evaluation index, that was *e*(*k*, *j*), was calculated as follows [26]:
    (5) $$e(k,j)=-\frac{1}{\ln N}\sum_{z=1}^{N}p(k,z,j)\ln p(k,z,j)$$
    where the value of *e*(*k*, *j*) ranges from 0 to 1, the smaller the value was, the greater the difference among the values of the index *j* in different regions was, and the more the information the index *j* transmitted, the larger the objective weight of the index *j* was.
    To consider the consistency among the initial weight of each index reflected by the information entropy, a fuzzy complementary judgment matrix *A*_o_*^k^* was constructed [26]:
    (6) $$A_{o}^{k}={\left(a_{ij}^{k}\right)}_{n_{k}\times n_{k}},a_{ij}^{k}=[1-e(k,i)]/[1-e(k,i)+1-e(k,j)],\;(k=1,2,3;i,j=1,2,\ldots ,n_{k})$$
    Similarly, according to the method for calculating subjective weight of index, the AGA-FAHP method was used to test and correct the consistency of *A*_o_*^k^*, and then the optimal fuzzy consistency judgment matrix *B*_o_*^k^* = (*b^k^_ij_*)*_n__k_*_×*n*_*_k_* and *w*_o_(*k*, *j*) was obtained by solving the following optimization problem:
    (7) $$\begin{array}{l} \min CIC(n_{k})=\sum_{i=1}^{n_{k}}\sum_{j=1}^{n_{k}}|b_{ij}^{k}-a_{ij}^{k}|/n_{k}^{2}+\sum_{i=1}^{n_{k}}\sum_{j=1}^{n_{k}}\left|0.5(n_{k}-1)[w_{o}(k,i)-w_{o}(k,j)]+0.5-b_{ij}^{k}\right|/n_{k}^{2}\\ s.t.\left\{ \begin{array}{l} b_{ii}^{k}=0.5,\;k=1,2,3;i=1,2,\ldots ,n_{k}\\ 1-b_{ji}^{k}=b_{ij}^{k}\in [a_{ij}^{k}-d,a_{ij}^{k}+d]\cap [0,1],\;k=1,2,3;i=1,2,\ldots ,n_{k};j=i+1,i+2,\ldots ,n_{k}\\ \sum_{j=1}^{n_{k}}w_{o}(k,j)=1.0,w_{o}(k,j)\in [0,1],\;k=1,2,3;j=1,2,\ldots ,n_{k} \end{array}\right. \end{array}$$
  - Combined weight. The combined weight of each index {*w*_c_(*k*, *j*)|*k* = 1, 2, 3; *j* = 1, 2, …, *n_k_*} should be as close as possible to the subjective weight *w*_s_(*k*, *j*) and objective weight *w*_o_(*k*, *j*). Therefore, according to the principle of the minimum relative entropy [35]:
    (8) $$\min F=\sum_{j=1}^{n_{k}}w_{c}(k,j)[\ln w_{c}(k,j)-\ln w_{s}(k,j)]+\sum_{j=1}^{n_{k}}w_{c}(k,j)[\ln w_{c}(k,j)-\ln w_{o}(k,j)]\;s.t.\sum_{j=1}^{n_{k}}w_{c}(k,j)=1,w_{c}(k,j)\in [0,1],k=1,2,3;j=1,2,\ldots ,n_{k}$$
    The problem in Equation (8) was solved by the Lagrange multiplier method:
    (9) $$w_{c}(k,j)=\frac{{\left[w_{s}(k,j)w_{o}(k,j)\right]}^{0.5}}{\sum_{j=1}^{n_{k}}{\left[w_{s}(k,j)w_{o}(k,j)\right]}^{0.5}},\;k=1,2,3;j=1,2,\ldots ,n_{k}$$
  - Comprehensive weight. The comprehensive weight of each index {*w*(*k*, *j*)|*k* = 1, 2, 3; *j* = 1, 2, …, *n_k_*} was calculated through multiplying the combined weight of each index by the subjective weight of the carrying subsystem the index belonged to as follows:
    (10) $$w(k,j)=w_{c}(k,j)w_{\mathrm{sub},k},\;k=1,2,3;j=1,2,\ldots ,n_{k}$$
    where *w*_sub,*k*_ is the subjective weight of *k*-th subsystem, which is obtained based on the AGA-FAHP method.
Step 4: Set pair analysis (SPA) is a systematic methodology to quantitatively describe and process the system uncertainty, proposed by scholar Zhao Keqin in 1989 [36]. In this theory, certainties and uncertainties were regarded as an integrated certain-uncertain system in which the certainty was divided into two parts of similarity and opposition, and the uncertainty was represented as difference. The core idea of SPA was firstly to construct a pair with two relevant sets in the integrated system, and then analyze the characteristics of the set pair and calculate corresponding connection numbers from those three aspects. Specifically in the evaluation of carrying capacity, the SPA was to realize the quantitative comparison between two sets (evaluation index and the corresponding grade criteria). The form of the connection number, *u*, was set as follows [37,38]:
  (11) u=a+bI+cJ
  where *a*, *b*, and *c* represent the degrees of similarity, difference, and opposition between evaluation index and corresponding grade criteria, all ranging from 0 to 1 and *a* + *b* + *c* = 1.
  First of all, the SPA was used to calculate the connection number of evaluation sample, that was *u*_1_, and it was obtained by the following formula [38]:
  (12) $$u_{1}(k,z)=a_{1}(k,z)+b_{1}(k,z)I+c_{1}(k,z)J$$
  (13) $$a_{1}{}_{z}=\sum_{k=1}^{3}a_{1}(k,z),\;b_{1}{}_{z}=\sum_{k=1}^{3}b_{1}(k,z),\;c_{1}{}_{z}=\sum_{k=1}^{3}c_{1}(k,z),u_{1z}=a_{1}{}_{z}+b_{1}{}_{z}I+c_{1}{}_{z}J$$
  where *u*_1_(*k*, *z*) is the ternary connection number of evaluation sample for the *k*-th subsystem in the region *z*; *u*_1*z*_ is the ternary connection number of evaluation sample for the region *z*; *a*_1_(*k*, *z*), *b*_1_(*k*, *z*), and *c*_1_(*k*, *z*) are the components of *u*_1_(*k*, *z*); *a*_1*z*_, *b*_1*z*_, and *c*_1*z*_ are the components of *u*_1*z*_; *I* is the difference coefficient, and *J* is the opposition coefficient. Furthermore, three components of *u*_1_(*k*, *z*) were calculated by the following equation:
  (14) $$a_{1}(k,z)=\sum_{j=1}^{n_{k1}}w(k,j),\;b_{1}(k,z)=\sum_{j=n_{k1}+1}^{n_{k1}+n_{k2}}w(k,j),\;c_{1}(k,z)=\sum_{j=n_{k1}+n_{k2}+1}^{n_{k1}+n_{k2}+n_{k3}}w(k,j)$$
  where *n_k_*_1_, *n_k_*_2_, and *n_k_*_3_ are the number of the index belonging to the evaluation grade of 1, 2 and 3 in the *k*-th subsystem, respectively, and they met the following conditions:
  (15) $$n_{k1}+n_{k2}+n_{k3}=n_{k},\;k=1,2,3,\;\sum_{k=1}^{3}n_{k}=n$$
Step 5: The SPA was used to calculate the connection number of evaluation index between the index *x*(*k*, *z*, *j*) and corresponding grade criterion *s*(*p*, *j*) for the *k*-th subsystem in the region *z*, that was *u*_2_(*k*, *z*, *j*). The SPA was to realize the quantitative comparison between *x*(*k*, *z*, *j*) and *s*(*p*, *j*) from the aspects of similarity, difference, and opposition; moreover, three components of ternary connection number *u*_2_(*k*, *z*, *j*) were calculated by Equations (16) and (17).
  For an index, the smaller its value was, the better the carrying situation was, that was a negative index, its ternary connection number was calculated by the following formula [38]:
  (16) $$a_{2}(k,z,j)=\left\{ \begin{array}{l} w(k,j),x(k,z,j)\leq s(1,j)\\ \frac{s(1,j)+s(2,j)-2x(k,z,j)}{s(2,j)-s(1,j)}\times w(k,j),s(1,j)<x(k,z,j)\leq \frac{s(1,j)+s(2,j)}{2}\\ 0,\;\frac{s(1,j)+s(2,j)}{2}<x(k,z,j)\leq s(2,j)\\ 0,\;x(k,z,j)>s(2,j) \end{array}\right.\;b_{2}(k,z,j)=\left\{ \begin{array}{l} 0,\;x(k,z,j)\leq s(1,j)\\ \frac{2x(k,z,j)-2s(1,j)}{s(2,j)-s(1,j)}\times w(k,j),s(1,j)<x(k,z,j)\leq \frac{s(1,j)+s(2,j)}{2}\\ \frac{2s(2,j)-2x(k,z,j)}{s(2,j)-s(1,j)}\times w(k,j),\frac{s(1,j)+s(2,j)}{2}<x(k,z,j)\leq s(2,j)\;\\ 0,\;x(k,z,j)>s(2,j) \end{array}\right.\;c_{2}(k,z,j)=\left\{ \begin{array}{l} 0,\;x(k,z,j)\leq s(1,j)\\ 0,\;s(1,j)<x(k,z,j)\leq \frac{s(1,j)+s(2,j)}{2}\\ \frac{2x(k,z,j)-s(1,j)-s(2,j)}{s(2,j)-s(1,j)}\times w(k,j),\frac{s(1,j)+s(2,j)}{2}<x(k,z,j)\leq s(2,j)\\ w(k,j),x(k,z,j)>s(2,j) \end{array}\right.$$
  Additionally, for an index, the larger its value was, the better the carrying situation was, that was a positive index, and its ternary connection number was calculated as follows [38]:
  (17) $$a_{2}(k,z,j)=\left\{ \begin{array}{l} w(k,j),x(k,z,j)\geq s(1,j)\\ \frac{2x(k,z,j)-s(1,j)-s(2,j)}{s(1,j)-s(2,j)}\times w(k,j),\frac{s(1,j)+s(2,j)}{2}\leq x(k,z,j)<s(1,j)\\ 0,\;s(2,j)\leq x(k,z,j)<\frac{s(1,j)+s(2,j)}{2}\\ 0,\;x(k,z,j)<s(2,j) \end{array}\right.\;b_{2}(k,z,j)=\left\{ \begin{array}{l} 0,\;x(k,z,j)\geq s(1,j)\\ \frac{2s(1,j)-2x(k,z,j)}{s(1,j)-s(2,j)}\times w(k,j),\frac{s(1,j)+s(2,j)}{2}\leq x(k,z,j)<s(1,j)\\ \frac{2x(k,z,j)-2s(2,j)}{s(1,j)-s(2,j)}\times w(k,j),s(2,j)\leq x(k,z,j)<\frac{s(1,j)+s(2,j)}{2}\\ 0,\;x(k,z,j)<s(2,j) \end{array}\right.\;c_{2}(k,z,j)=\left\{ \begin{array}{l} 0,\;x(k,z,j)\geq s(1,j)\\ 0,\;\frac{s(1,j)+s(2,j)}{2}\leq x(k,z,j)<s(1,j)\\ \frac{s(1,j)+s(2,j)-2x(k,z,j)}{s(1,j)-s(2,j)}\times w(k,j),s(2,j)\leq x(k,z,j)<\frac{s(1,j)+s(2,j)}{2}\\ w(k,j),x(k,z,j)<s(2,j) \end{array}\right.$$
  where *a*_2_(*k*, *z*, *j*), *b*_2_(*k*, *z*, *j*), and *c*_2_(*k*, *z*, *j*) are the components of *u*_2_(*k*, *z*, *j*); *s*(1, *j*) and *s*(2, *j*) are the upper limit values of grade of 1 and 2 for the index *j*; for the negative index, there is *s*(1, *j*) < *s*(2, *j*), and for the positive index, there is *s*(1, *j*) > *s*(2, *j*).
  The connection number of each evaluation index was obtained by its three components:
  (18) $$u_{2}(k,z,j)=a_{2}(k,z,j)+b_{2}(k,z,j)I+c_{2}(k,z,j)J$$
  (19) $$a_{2}(k,z)=\sum_{j=1}^{n_{k}}a_{2}(k,z,j),\;b_{2}(k,z)=\sum_{j=1}^{n_{k}}b_{2}(k,z,j),\;c_{2}(k,z)=\sum_{j=1}^{n_{k}}c_{2}(k,z,j),\;u_{2}(k,z)=a_{2}(k,z)+b_{2}(k,z)I+c_{2}(k,z)J$$
  (20) $$a_{2}{}_{z}=\sum_{k=1}^{3}a_{2}(k,z),\;b_{2}{}_{z}=\sum_{k=1}^{3}b_{2}(k,z),\;c_{2}{}_{z}=\sum_{k=1}^{3}c_{2}(k,z),\;u_{2z}=a_{2}{}_{z}+b_{2}{}_{z}I+c_{2}{}_{z}J$$
  where *u_2_*(*k*, *z*) is the ternary connection number of index for the *k*-th subsystem in the region *z*; *u_2z_* is the ternary connection number of index for the region *z*; *a*_2_(*k*, *z*), *b*_2_(*k*, *z*), and *c*_2_(*k*, *z*) are the components of *u*_2_(*k*, *z*); and *a*_2*z*_, *b*_2*,*_ and *c*_2*z*_ are the components of *u*_2*z*_.
Step 6: Calculating the average connection number for the region *z*, *u_z_*. The average connection numbers obtained by Equation (21) could be regarded as a distribution with the similarity, difference and opposition items, they were *a*, *b*, *c*, which should be as close as possible to *a*_1_, *b*_1_, *c*_1_ and *a*_2_, *b*_2_, *c*_2_. Therefore, according to the principle of the minimum relative entropy [35]:
  (21) $$a(k,z)=\frac{{\left(a_{1}(k,z)a_{2}(k,z)\right)}^{0.5}}{{\left(a_{1}(k,z)a_{2}(k,z)\right)}^{0.5}+{\left(b_{1}(k,z)b_{2}(k,z)\right)}^{0.5}+{\left(c_{1}(k,z)c_{2}(k,z)\right)}^{0.5}},\;a_{z}=\frac{{\left(a_{1}{}_{z}a_{2}{}_{z}\right)}^{0.5}}{{\left(a_{1}{}_{z}a_{2}{}_{z}\right)}^{0.5}+{\left(b_{1}{}_{z}b_{2}{}_{z}\right)}^{0.5}+{\left(c_{1}{}_{z}c_{2}{}_{z}\right)}^{0.5}}\;b(k,z)=\frac{{\left(b_{1}(k,z)b_{2}(k,z)\right)}^{0.5}}{{\left(a_{1}(k,z)a_{2}(k,z)\right)}^{0.5}+{\left(b_{1}(k,z)b_{2}(k,z)\right)}^{0.5}+{\left(c_{1}(k,z)c_{2}(k,z)\right)}^{0.5}},\;b_{z}=\frac{{\left(b_{1}{}_{z}b_{2}{}_{z}\right)}^{0.5}}{{\left(a_{1}{}_{z}a_{2}{}_{z}\right)}^{0.5}+{\left(b_{1}{}_{z}b_{2}{}_{z}\right)}^{0.5}+{\left(c_{1}{}_{z}c_{2}{}_{z}\right)}^{0.5}}\;c(k,z)=\frac{{\left(c_{1}(k,z)c_{2}(k,z)\right)}^{0.5}}{{\left(a_{1}(k,z)a_{2}(k,z)\right)}^{0.5}+{\left(b_{1}(k,z)b_{2}(k,z)\right)}^{0.5}+{\left(c_{1}(k,z)c_{2}(k,z)\right)}^{0.5}},\;c_{z}=\frac{{\left(c_{1}{}_{z}c_{2}{}_{z}\right)}^{0.5}}{{\left(a_{1}{}_{z}a_{2}{}_{z}\right)}^{0.5}+{\left(b_{1}{}_{z}b_{2}{}_{z}\right)}^{0.5}+{\left(c_{1}{}_{z}c_{2}{}_{z}\right)}^{0.5}}\;u(k,z)=a(k,z)+b(k,z)I+c(k,z)J,\;u_{z}=a_{z}+b_{z}I+c_{z}J$$
Step 7: To improve the accuracy of evaluation result, the level eigenvalue method was used to calculate the grade of carrying capacity (*h_z_*) according to the following formula [32]:
  (22) $$h_{z}=a_{z}+2b_{z}+3c_{z}$$
  Meanwhile, to compare with the results obtained by the level eigenvalue method, the attribute recognition method was also used to calculate the grade of capacity (*g_z_*) [32]:
  (23) $$g_{z}=\left\{ \begin{array}{l} 1,\;a_{z}>\lambda \\ 2,\;a_{z}\leq \lambda \;\mathrm{and}\;a_{z}+b_{z}>\lambda \\ 3,\;a_{z}+b_{z}\leq \lambda \;\mathrm{and}\;a_{z}+b_{z}+c_{z}>\lambda \end{array}\right.$$
  where *λ* represents the confidence level, usually ranging from 0.50 to 0.70. The larger the value of *λ* is, the more reliable the evaluation result tends to be.
Step 8: The set pair potential (SPP) [39] was used to identify the vulnerability factors for the regional water resources carrying. The previous construction method of the SPP based on division, that was *s_f_*_1_(*u*) = *a*/*c*, tended to be unstable when the value of *c* was small. For example, 0.9000 + 0.0990*I* + 0.0010*J* and 0.9000 + 0.0999*I* + 0.0001*J*, the difference between these two connection numbers was small; however, their values of *s_f_*_1_(*u*) were 900 and 9000. In addition, the SPP based on exponent, that was *s_f_*_2_(*u*) = e*^a^*^–*c*^, changed the magnitude relationship between the degrees of similarity and opposition. Therefore, the SPP based on subtraction, that was *s_f_*(*u*), was proposed in this study as follows:
  (24) $$s_{f}(u)=a+ab-(c+cb)=(a-c)(1+b)$$
  In Equation (24), *b* belonging to the uncertain term was allocated based on the ratio value method. If *b* was fully assigned to *a* or *c*, the corresponding optimistic SSP *s_fa_*(*u*) = (*a* + *b*) – *c* or pessimistic SSP *s_fc_*(*u*) = *a* − (*c* + *b*) were obtained. It was obvious that the *s_f_*(*u*) ranged from −1.0 to 1.0 and *s_fc_*(*u*) ≤ *s_f_*(*u*) ≤ *s_fa_*(*u*). Moreover, according to the principle of equal division, the *s_f_*(*u*) was divided into five levels, there were inverse potential (−1.0 ≤ *s_f_*(*u*) < −0.6), partial inverse potential (−0.6 ≤ *s_f_*(*u*) < −0.2), symmetrical potential (−0.2 ≤ *s_f_*(*u*) ≤ 0.2), partial identical potential (0.2 < *s_f_*(*u*) ≤ 0.6), and identical potential (0.6 < *s_f_*(*u*) ≤ 1.0). The index belonging to the partial inverse or inverse potential was the main factor deteriorating the carrying status of regional water resources, which was identified as the vulnerability index. This index was the primary target for the regulation of control of carrying capacity.

## 3. Evaluating and Diagnosing Water Resources Carrying Capacity in Anhui Province

The evaluation and diagnosis approach was applied to 16 cities in Anhui Province, China. To explore the various driving mechanism of water resources carrying in different districts, these cities were divided into three areas referring to the urban planning of Anhui Province from 2011 to 2030, as well as the regions the Yangtze River and Huai River flowed through. They were the northern Anhui area along the north of Huai River (Huaibei, Bozhou, Suzhou, Bengbu, Fuyang, and Huainan, six cities), the Middle Anhui area between Huai River and the Yangtze River (Hefei, Chuzhou, Lu’an and Anqing, four cities) and the Southern Anhui area along the south of the Yangtze River (Ma’anshan, Wuhu, Xuancheng, Tongling, Chizhou and Huangshan, six cities), respectively.

### 3.1. Evaluation Index System and Index Weight

Based on the systematic analysis of carrying process, the natural and social conditions of water resources and the previous research [1,2,10,40], the evaluation index system consisting of three carrying subsystems (support force, pressure force and regulation force), fifteen evaluation indices (*X*_1_–*X*_15_) and corresponding grade criteria in Anhui Province were constructed (Table 1). Meanwhile, according to the statistics of the Anhui Province Statistical Yearbook (2012–2016) and the Anhui Province Water Resources Bulletin (2011–2015), the evaluation samples were obtained.

Experts were invited to compare the importance of three carrying subsystems and fifteen indices in Table 1 in pairs, and then the following four fuzzy complementary judgment matrices were obtained as:

$$A_{s}=\left[\begin{array}{ccc} 0.5 & 0.5 & 0.7\\ 0.5 & 0.5 & 0.7\\ 0.3 & 0.3 & 0.5 \end{array}\right]\;A_{s}^{1}=\left[\begin{array}{cccc} 0.5 & 0.5 & 0.6 & 0.9\\ 0.5 & 0.5 & 0.6 & 0.9\\ 0.4 & 0.4 & 0.5 & 0.8\\ 0.1 & 0.1 & 0.2 & 0.5 \end{array}\right],\;A_{s}^{2}=\left[\begin{array}{cccccc} 0.5 & 0.2 & 0.4 & 0.2 & 0.4 & 0.2\\ 0.8 & 0.5 & 0.6 & 0.5 & 0.6 & 0.5\\ 0.6 & 0.4 & 0.5 & 0.4 & 0.4 & 0.4\\ 0.8 & 0.5 & 0.6 & 0.5 & 0.6 & 0.5\\ 0.6 & 0.4 & 0.6 & 0.4 & 0.5 & 0.4\\ 0.8 & 0.5 & 0.6 & 0.5 & 0.6 & 0.5 \end{array}\right],\;A_{s}^{3}=\left[\begin{array}{ccccc} 0.5 & 0.6 & 0.8 & 0.6 & 0.9\\ 0.4 & 0.5 & 0.7 & 0.55 & 0.6\\ 0.2 & 0.3 & 0.5 & 0.4 & 0.6\\ 0.4 & 0.45 & 0.6 & 0.5 & 0.8\\ 0.1 & 0.4 & 0.4 & 0.2 & 0.5 \end{array}\right]$$

Substituting *A*_s_ into Equation (3), where *d* was 0.2, and then applying the AGA to solve the optimization problem, the subjective weights of three carrying subsystems were calculated (Table 2).

- The *CIC* of *A*_s_ was lower than 0.20, indicating that *A*_s_ had a satisfactory consistency, and the calculated subjective weights of the subsystems were acceptable.
- The weight of the carrying regulation force subsystem was the smallest. It reflected that the carrying status of water resources in Anhui Province was mainly determined by the balance between the support force and the pressure force, the function of the regulation force that just played a role of adjusting this balance was relatively small. This was in accord with the mechanism of regional water resources carrying.
- Similarly, the *CIC* of these three matrices for calculating the subjective weight of the index were all less than 0.20. Therefore, the obtained subjective weight of each index was acceptable.
- Substituting the annual average value of each index from 2011 to 2015 in 16 cities into Equations (4), (5), and (6) in sequence, *A*_o_^1^, *A*_o_^2^, and *A*_o_^3^ were constructed. Next, these matrices were substituted into Equation (7), where *d* was 0.2, and then using the AGA-FAHP method to calculate the objective weights of fifteen indices (Table 2).
- The *CIC* of these three matrices were all less than 0.20; therefore, the objective weights of fifteen indices were all acceptable.
- The calculated subjective and objective weights of each index were basically consistent. For example, the subjective and objective weights of *X*_1_ were 0.33 and 0.38, which were both the largest in the support force subsystem. In addition, the subjective and objective weights of *X*_5_ were 0.10 and 0.06, which were both the smallest in the pressure force subsystem. However, there were certain differences between the two types of weight for some indices. The subjective weight of *X*_15_ was only 0.11, which was the minimum value in the regulation force subsystem, while its objective result was 0.24. Therefore, to scientifically determine the index weight, it was reasonable and necessary to combine the subjective and objective weights.
  $$A_{o}^{1}=\left[\begin{array}{l} 0.50\;0.81\;0.73\;0.75\\ 0.19\;0.50\;0.39\;0.41\\ 0.27\;0.61\;0.50\;0.52\\ 0.25\;0.59\;0.48\;0.50 \end{array}\right],\;A_{o}^{2}=\left[\begin{array}{l} 0.50\;0.07\;0.04\;0.05\;0.19\;0.07\\ 0.93\;0.50\;0.33\;0.42\;0.75\;0.48\\ 0.96\;0.67\;0.50\;0.59\;0.86\;0.65\\ 0.95\;0.59\;0.41\;0.50\;0.81\;0.56\\ 0.81\;0.25\;0.14\;0.19\;0.50\;0.23\\ 0.93\;0.52\;0.35\;0.44\;0.77\;0.50 \end{array}\right],\;A_{o}^{3}=\left[\begin{array}{l} 0.50\;0.72\;1.00\;0.58\;0.61\\ 0.28\;0.50\;0.99\;0.36\;0.39\\ 0.00\;0.01\;0.50\;0.00\;0.00\\ 0.42\;0.64\;1.00\;0.50\;0.53\\ 0.39\;0.61\;1.00\;0.47\;0.50 \end{array}\right]$$

Substituting the subjective and objective weights of each index into Equation (9), the combined weight was calculated. Then, multiplying the combined weight of each index by the subjective weight of the carrying subsystem the index belonged to (Equation (10)), the comprehensive weight of each index was obtained (Table 2).

The comprehensive weight of *X*_1_ (0.15) was the largest among fifteen indices, followed by *X*_2_ and *X*_3_, these three indices all belonged to the support force subsystem. This indicated that the impact of support force on the final carrying status was relatively great, which was regarded as the supply item in the process of water resources carrying. Moreover, the amount of regional water resources and population were the key factors for determining the size of the support force in Anhui.

### 3.2. Temporal and Spatial Distribution of Carrying Capacity in Different Areas

Substituting the samples and comprehensive weight of each index into Equations (12)–(14), the connection number of evaluation sample (*u*_1_) was calculated. Meanwhile, substituting the above data into Equations (16) (or Equation (17)), and (18)–(20), the connection number of evaluation index (*u*_2_) was obtained. The average connection number (*u*) was acquired by Equation (21). Next, the evaluation grade value of water resources carrying capacity was calculated based on the level of the eigenvalue and attribute recognition methods (λ was 0.55) by Equations (22) and (23), and the larger the value was, the worse the carrying status was. Furthermore, the carrying status was divided into five levels through the grade value obtained by the level eigenvalue method; they were loadable status (1.001 ≤ grade value ≤ 1.750), critical status (1.751 ≤ grade value ≤ 2.000), slight overloaded status (2.001 ≤ grade value ≤ 2.250), moderate overloaded status (2.251 ≤ grade value ≤ 2.500), and serious overloaded status (2.501 ≤ grade value ≤ 3.000). Similarly, the status was divided into three levels according to the grade value based on the attribute identification method; they were loadable status (grade value = 1), critical status (grade value = 2) and overloaded status (grade value = 3). The average grade values of carrying capacity from 2011 to 2015 in 16 cities are shown in Table 3.

- Among the three types of connection numbers, *a* was the largest in Huangshan, *b* was largest in Lu’an and Anqing, and *c* was the largest in Fuyang. This indicated that, for the same component in different types of connection numbers, the results they expressed were consistent; therefore, it was more reliable to use the average connection number to reflect the actual temporal and spatial variations of carrying status.
- According to the grade values calculated based on the average connection number and level eigenvalue method, there were 11 cities in the overloaded status and merely three cities in the loadable status. This indicated that the overall situation of water resources carrying in Anhui Province was quite severe in recent years, and it was necessary to explore its carrying mechanism and diagnose the main vulnerability factors for improving its carrying condition.
- Among 11 cities whose carrying status were overloaded, there were six, two, and three cities in Northern, Middle, and Southern Anhui which accounted for 100%, 50%, and 50% of the total cities in each area. The overloaded situation in Fuyang was the most serious; its grade value reached 2.45. However, the three cities with loadable status were all located in the southern part. The average grade values in Northern, Middle, and Southern Anhui were 2.36, 1.97, and 1.93. This reflected that there were large differences in the carrying status among various areas; the carrying condition in Southern and Middle Anhui were obviously better than that in the northern area, and the southern and middle parts were at the edge of the critical status to the slight overloaded situation, while the northern area was in the moderate overloaded level.

**Figure 2:**
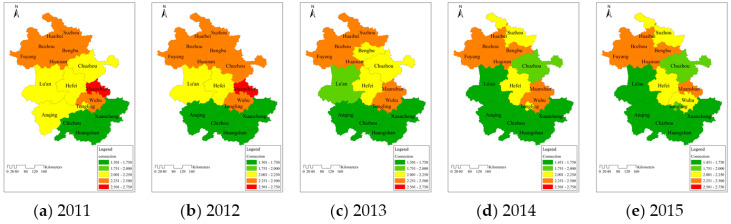
Temporal and spatial distribution of water resources carrying capacity among 16 cities in Anhui Province during (**a**) 2011; (**b**) 2012; (**c**) 2013; (**d**) 2014 and (**e**) 2015.

To analyze the mechanism of water resource carrying and the reasons for the variation of carrying status in each area, the grade values to which the average connection number corresponded in 16 cities from 2011 to 2015 are shown in Figure 2.

- From 2011 to 2015, most cities were in the overloaded state (2.001 ≤ grade value ≤ 3.000), and they were distributed mainly in the middle and northern areas along the north of the Yangtze River. This indicated that the overall carrying situation in Anhui Province was still very serious. In addition, the differences in carrying status among different areas were obvious; its distribution was that the condition in Southern Anhui was better than that in the middle part, and in the northern area was relatively poor. Therefore, the carrying capacity of water resources may be influenced by the geographical attributes in study region.
- Based on the variations of the carrying status in the three areas from 2011 to 2015, the number of cities in the overloaded status was reduced by four, and there were three and one cities in the middle and southern areas. The grade values of carrying capacity in Northern, Middle, and Southern Anhui declined by 0.05, 0.31, and 0.21. It reflected that although the carrying condition in Anhui Province was severe, its overall development tended to be better. However, the overloaded status in the northern part did not essentially change, while the situation in the middle and southern areas improved markedly. Therefore, it was crucial to identify the reasons for the variations of carrying situation in each area for regulating the regional water resources.

### 3.3. Temporal and Spatial Distribution of Carrying Subsystems in Different Areas

To deeply analyze the reasons for the variation of carrying status in each area, the key driving factors in different areas were discussed from the perspective of the carrying subsystem.

#### 3.3.1. Temporal and Spatial Distribution of Carrying Support Force

**Figure 3:**
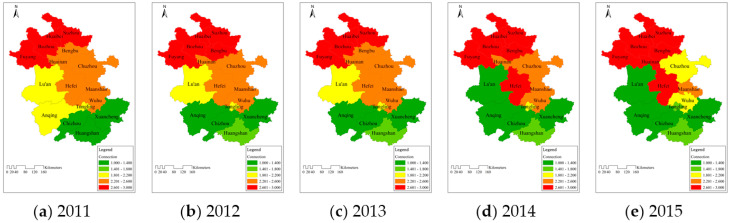
Temporal and spatial distribution of water resources carrying support force among 16 cities in Anhui Province during (**a**) 2011; (**b**) 2012; (**c**) 2013; (**d**) 2014 and (**e**) 2015.

The grade values of water resources carrying support force in 16 cities from 2011 to 2015 are shown in Figure 3, and the larger the value is, the weaker the support force.

- The differences in support force among various areas were obvious, and its distribution was that the support force in Southern Anhui was stronger than that in the middle area, and that in the northern part was relatively weak. The average grade values from 2011 to 2015 in Northern, Middle, and Southern Anhui were 2.85, 2.02, and 1.72. This was consistent with the results in Figure 2 and Table 3. Therefore, the difference in the support force was the main factor resulting in the large difference in the final carrying capacity among the different areas.
- The overall situation of support force in Northern Anhui did not improve effectively, its grade value basically ranged from 2.601 to 3.000. This indicated that the support force in this area had been at a low level, which was the vital element to obstruct its improvement of carrying capacity. The grade values in the middle and southern areas in 2015 were 0.45 and 0.21 lower than those in 2011. This was in agreement with their improved carrying situations in recent years; therefore, the increased support force made the carrying status better.

#### 3.3.2. Temporal and Spatial Distribution of Carrying Pressure Force

**Figure 4:**
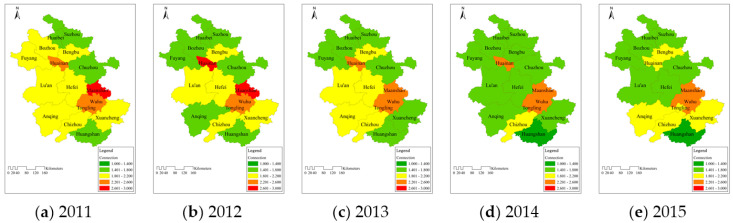
Temporal and spatial distribution of water resources carrying pressure force among 16 cities in Anhui Province during (**a**) 2011; (**b**) 2012; (**c**) 2013; (**d**) 2014 and (**e**) 2015.

The grade values of water resources carrying pressure force in 16 cities from 2011 to 2015 are shown in Figure 4, and the larger the value is, the greater the pressure force.

- The pressure force in Southern Anhui was greater than those in the middle and northern parts, and the differences were relatively small compared with those in support force and tended to decline. The average grade values from 2011 to 2015 in Southern, Middle, and Northern Anhui were 2.06, 1.85, and 1.84. This was not consistent with the results of carrying capacity distribution among these three areas. It was mainly due to small differences in the pressure force, in that they could not cause significant variation of carrying status.
- The grade values of pressure force in the northern, middle, and southern areas in 2015 were 0.18, 0.21, and 0.17 lower than those in 2011. It reflected that the pressure force in Anhui Province had been relieved in recent years; the effect in the middle part was relatively obvious. This explained the reason for the improved carrying situation from the perspective of pressure force

#### 3.3.3. Temporal and Spatial Distribution of Carrying Regulation Force

**Figure 5:**
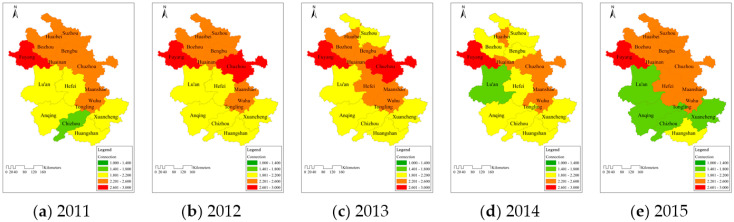
Temporal and spatial distribution of water resources carrying regulation force among 16 cities in Anhui Province during (**a**) 2011; (**b**) 2012; (**c**) 2013; (**d**) 2014 and (**e**) 2015.

The grade values of water resources carrying regulation force in 16 cities from 2011 to 2015 are shown in Figure 5, and the larger the value is, the weaker the regulation force.

- The regulation force in the southern and middle areas were stronger than that in the northern part, and the differences were smaller than those in support force, but they showed the increasing tendency. The average grade values from 2011 to 2015 in Northern, Middle, and Southern Anhui were 2.40, 2.10, and 2.10. This was in agreement with their distributions of carrying capacity in Figure 2. It indicated that the differences in regulation force would affect the final carrying status, but this effect was smaller than that of support force.
- The overall regulation force in Northern Anhui tended to be stronger, but its carrying situation had not improved in recent years. It reflected that the influence of regulation force on carrying capacity was small; the regulation force was not the main carrying driving factor in this area. The grade values of regulation force in Middle and Southern Anhui in 2015 were decreased by 0.24 and 0.29 compared with 2011. This showed that their regulation force were strengthened, and this was consistent with their improved carrying status. Therefore, the enhanced regulation force was the primary cause of the increased carrying capacity in these two areas.

Combining with the grade values of carrying capacity and also support force, pressure force and regulation force from 2011 to 2015, the causes of variations in carrying status were analyzed.

- The contribution rates of support force, pressure force and regulation force to carrying capacity in Middle Anhui were 41.06% (2.02 (the average grade value of support force from 2011 to 2015) × 0.4 (the weight of support force subsystem) ÷ 1.97 (the average grade value of carrying capacity) × 100%), 37.60% (1.85 × 0.4 ÷ 1.97 × 100%), and 21.34% (2.10 × 0.2 ÷ 1.97 × 100%). The influences of three subsystems on final carrying status were basically uniform; therefore, the reasons for carrying situations in the other two areas were discussed based on the middle part.
- The average grade value of carrying capacity from 2011 to 2015 in Northern Anhui was 0.39 larger than that in the middle area, and those of support force, pressure force, and regulation force were 0.83, −0.01, and 0.30 larger; therefore, their contribution rates were 85.57%, −1.03%, and 15.38%. This indicated that, for Northern Anhui, the major factor that obstructed the development of the carrying capacity was its deficient support force. Similarly, the strong support force guaranteed the good carrying condition in the southern area.
- In Northern Anhui, the grade value of the carrying capacity in 2015 was 0.05 lower than that in 2011, and those of the support force, pressure force, and regulation force were −0.03, 0.18, and −0.05 lower. Therefore, their contribution rates were −24.00%, 144.00%, and −20.00%, respectively. This reflected that the reduced pressure force was the main reason for its slight improvement of the capacity, and the weakened support force and regulation force accounted for its long-term overloaded situation. Similarly, the strengthened support force was the primary cause of the improvement of carrying status in the middle area; the enhanced support force and reduced pressure force mainly made the condition better in the southern part.

### 3.4. Set Pair Potential Based on Subtraction of Evaluation Index

It was necessary to further diagnose and identify the carrying vulnerability index based on the SPP for the regulation of regional water resources carrying capacity. The average SPP of each index from 2011 to 2015 in different areas were calculated according to Equation (24), as shown in Table 4.

- There were three, two, and two identical potential indices, and five, three, and one inverse potential indices in Northern, Middle, and Southern Anhui. This was consistent with the distribution of carrying status among these three areas. Similarly, the numbers of identical and inverse potential indices in the three carrying subsystems were also in agreement with their spatial distributions. Therefore, the number of identical and inverse potential index in a certain region could basically reflect its relatively good or poor carrying situation; moreover, the less the identical potential index and the more the inverse potential index were, the worse the carrying condition was, and vice versa.
- For Northern Anhui, *X*_1_, *X*_2_, *X*_4_, *X*_8_, and *X*_15_ were carrying vulnerability indices, and there were three, one, and one indices in the support force, pressure force, and regulation force subsystems. It indicated that the primary factor limited its carrying capacity was the insufficient support force. This was consistent with the analysis on the contribution rate of the support force to the carrying capacity in this area. From the view of the index, its long-term overloaded status was mainly caused by the fewer water resources, which restricted the support force; therefore, its carrying situation may be improved through water diversion. Similarly, deficient ecological water consumption and limited water-saving irrigation area seriously obstructed the promotion of the carrying capacity in the middle area; the long-term good carrying status in the southern part was due largely to its relatively abundant water resources and relatively small population size. Therefore, the water diversion project from the Yangtze River to the Huai River was a great strategy for enhancing the whole carrying level in Anhui Province.

**Figure 6:**
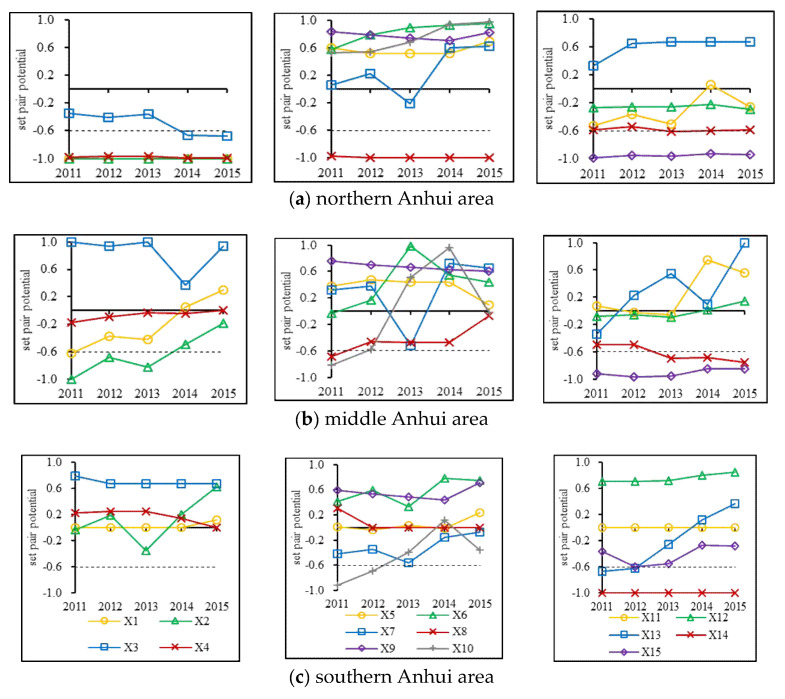
Set pair potential based on subtraction of each index in three carrying subsystems from 2011 to 2015 among different areas in Anhui Province.

Based on the SPP of each index among three areas from 2011 to 2015 in Figure 6, the reasons for the variations in each carrying subsystem were discussed from the perspective of the index.

- For Northern Anhui, the SPP of *X*_1_, *X*_2_, and *X*_4_ were basically in the maximum inverse potential status, and that of *X_3_* backed the inverse potential from the partial inverse potential in 2014. It reflected that the support force was weakened, and the primary reason was the decreased water supply per capita. This was in agreement with the temporal variation of the support force in this area. Similarly, the pressure force was reduced, and this was due mostly to the control of industrial and agricultural water consumption. In addition, the overall regulation force was strengthened, and the major causes were the increased wastewater treatment level and reduced water resources utilization in recent years.
- In the middle part, the support force was enhanced, and the main reasons were the increased water resources and vegetation coverage. The controlled population size and agricultural water consumption resulted in the decreased pressure force; the reduced water resources utilization and increased wastewater treatment level made the regulation force stronger.
- For the southern area, the support force was strengthened by the increased water resources; the reason for the decreased pressure force was the reduced agricultural water consumption; and the regulation force was promoted by the improved wastewater treatment level.

## 4. Conclusions

In this paper, an index system and corresponding grade criteria for evaluating the carrying capacity of regional water resources were constructed from the aspects of three carrying subsystems. Meanwhile, a method that combined the information entropy and an improved fuzzy analytic hierarchy process was used to calculate the objective weight of the index. Then, a quantitative evaluation model was established by applying set pair analysis and a set pair potential based on subtraction was proposed to diagnose the carrying vulnerability factors. Furthermore, an empirical research was carried out in Anhui Province, and the following main conclusions were obtained:

(1) The carrying situations in Southern and Middle Anhui were obviously superior to that in the northern area. Moreover, the support force in Southern Anhui was stronger than that in the middle area, and in the northern part was relatively weak. The pressure force in Southern Anhui was greater than those in the middle and northern parts, the differences were relatively small compared with those in support force and tended to decline. Furthermore, the regulation force in the southern and middle areas were stronger than that in the northern part, and the differences were smaller than those in support force, but they showed the increasing tendency. In addition, although the carrying condition in Anhui Province was severe, its overall development tended to be improved. However, the overload status in the northern part did not essentially change, while the situation in the middle and southern areas improved markedly.
(2) For Northern Anhui, the main factor obstructed the development of capacity was its deficient support force; the stronger support force guaranteed the good carrying condition in the southern part. Furthermore, the reduced pressure force was the main reason for its slight improvement of capacity, and the weakened support force and regulation force accounted for its long-term overloaded situation in Northern Anhui. The strengthened support force was the primary cause of the improvement of carrying status in the middle area; the enhanced support force and reduced pressure force mainly made the carrying condition better in the southern part.
(3) For Northern Anhui, its long-term overloaded status was mainly caused by the fewer water resources. Moreover, the deficient ecological water consumption and limited water-saving irrigation area seriously obstructed the promotion of capacity in the middle area. The long-term loadable status in the southern part was due largely to its relatively abundant water resources and small population size. Therefore, the water diversion project from the Yangtze River to the Huai River was a great strategy for enhancing the whole carrying level in Anhui Province.
(4) For Northern Anhui, the support force was weakened, and the primary reason was the decreased water supply per capita; the pressure force was reduced, and it was due mostly to the control of industrial and agricultural water consumption; in addition, the overall regulation force was strengthened, and the major causes were the increased wastewater treatment level and reduced water resources utilization in recent years. However, in the middle part, the support force was enhanced, and the main reasons were the increased water resource and vegetation coverage; the controlled population size and agricultural water consumption resulted in the decreased pressure force; and the reduced water resources utilization and increased wastewater treatment level made the regulation force stronger. For the southern area, the support force was strengthened by the increased water resources; the reason for the decreased pressure force was the reduced agricultural water consumption; and the regulation force was promoted by the improved wastewater treatment level.

## Figures and Tables

**Table 1:** Index system and corresponding grade criteria for evaluating the carrying capacity of water resources in Anhui Province.

Water Resources Carrying Capacity System	Evaluation Index	Water Resources Carrying Grade Criterion		
		Loadable Status	Critical Status	Overloaded Status
water resources carrying support force subsystem	*X*_1_ water resources per capita (m^3^/person)	>1670	[1000, 1670]	<1000
	*X*_2_ production modulus of water resources (10^4^ m^3^/km^2^)	>80	[50, 80]	<50
	*X*_3_ water supply per capita (m^3^/(person · year))	>450	[350, 450]	<350
	*X*_4_ rate of vegetation coverage (%)	>40	[25, 40]	<25
water resources carrying pressure force subsystem	*X*_5_ daily domestic water consumption per capita (L/(person · day))	<70	[70, 180]	>180
	*X*_6_ water consumption per 10^4^ yuan (m^3^/10^4^ yuan)	<100	[100, 400]	>400
	*X*_7_ water consumption per 10^4^ yuan of value-added by industry (m^3^/10^4^ yuan)	<50	[50, 200]	>200
	*X*_8_ density of population (person/km^2^)	<200	[200, 500]	>500
	*X*_9_ rate of urbanization (%)	<50	[50, 80]	>80
	*X*_10_ water consumption per mu for agricultural irrigation (m^3^/mu)	<250	[250, 400]	>400
water resources carrying regulation force subsystem	*X*_11_ rate of water resources utilization (%)	<40	[40, 70]	>70
	*X*_12_ gross domestic product per capita (10^4^ yuan/person)	>24840	[6624, 24,840]	<6624
	*X*_13_ rate of urban wastewater treatment (%)	>95	[90, 95]	<90
	*X*_14_ rate of water-saving irrigation area (%)	>60	[20, 60]	<20
	*X*_15_ rate of ecological water consumption (%)	>5	[1, 5]	<1

**Table 2:** Weights of indices for evaluating the carrying capacity of water resources in Anhui Province and the consistency index coefficients (CIC) of the corresponding judgment matrices.

Water Resources Carrying Capacity System	Improved Fuzzy Analytic Hierarchy Process		Evaluation Index	Improved Fuzzy Analytic Hierarchy Process		Improved Entropy Weight Method		Combined Weight	Comprehensive Weight
	Weight of Subsystem	CIC		Subjective Weight	CIC	Objective Weight	CIC		
support force subsystem	0.40	0.000	*X*_1_ water resources per capita (m^3^/person)	0.33	0.012	0.38	0.012	0.36	0.15
			*X*_2_ production modulus of water resources (10^4^ m^3^/km^2^)	0.33		0.17		0.25	0.10
			*X*_3_ water supply per capita (m^3^/(person · year))	0.26		0.23		0.25	0.10
			*X*_4_ rate of vegetation coverage (%)	0.08		0.22		0.14	0.05
pressure force subsystem	0.40		*X*_5_ daily domestic water consumption per capita (L/(person · day))	0.10	0.031	0.06	0.055	0.08	0.03
			*X*_6_ water consumption per 10^4^ yuan (m^3^/10^4^ yuan)	0.20		0.19		0.20	0.08
			*X*_7_ water consumption per 10^4^ yuan of value-added by industry (m^3^/10^4^ yuan)	0.15		0.23		0.19	0.08
			*X*_8_ density of population (person/km^2^)	0.20		0.22		0.21	0.08
			*X*_9_ rate of urbanization (%)	0.16		0.10		0.13	0.05
			*X*_10_ water consumption per mu for agricultural irrigation (m^3^/mu)	0.20		0.20		0.20	0.08
regulation force subsystem	0.20		*X*_11_ rate of water resources utilization (%)	0.28	0.038	0.28	0.051	0.29	0.06
			*X*_12_ gross domestic product per capita (10^4^ yuan/person)	0.23		0.18		0.21	0.04
			*X*_13_ rate of urban wastewater treatment (%)	0.16		0.05		0.09	0.02
			*X*_14_ rate of water-saving irrigation area (%)	0.22		0.25		0.24	0.05
			*X*_15_ rate of ecological water consumption (%)	0.11		0.24		0.17	0.03

**Table 3:** Three types of connection numbers and corresponding evaluation grade values of water resource carrying capacity based on the average value of evaluation samples from 2011 to 2015 in Anhui Province.

Evaluation City	Connection Number of Evaluation Sample					Connection Number of Evaluation Index					Average Connection Number				
	*u*_1_ = *a*_1_+*b*_1_*I*+*c*_1_*J*			Grade Value		*u*_2_ = *a*_2_ + *b*_2_*I* + *c*_2_*J*			Grade Value		*u* = *a* + *bI* + *cJ*			Grade Value	
	*a*_1_	*b*_1_	*c*_1_	LE ^1^	AR ^2^	*a*_2_	*b*_2_	*c*_2_	LE	AR	*a*	*b*	*c*	LE	AR
Huaibei	0.24	0.20	0.56	2.33	3	0.29	0.12	0.59	2.30	3	0.26	0.15	0.58	2.32	3
Bozhou	0.16	0.27	0.57	2.41	3	0.28	0.11	0.61	2.33	3	0.22	0.18	0.60	2.39	3
Suzhou	0.19	0.34	0.47	2.28	3	0.29	0.14	0.57	2.27	3	0.24	0.23	0.53	2.29	3
Bengbu	0.17	0.38	0.45	2.28	2	0.25	0.23	0.52	2.27	3	0.21	0.30	0.49	2.29	3
Fuyang	0.15	0.28	0.58	2.43	3	0.24	0.10	0.66	2.43	3	0.19	0.17	0.64	2.45	3
Huainan	0.13	0.32	0.55	2.41	3	0.19	0.22	0.58	2.39	3	0.16	0.27	0.57	2.41	3
Hefei	0.28	0.27	0.45	2.17	2	0.32	0.17	0.51	2.20	3	0.30	0.22	0.48	2.18	3
Chuzhou	0.23	0.44	0.33	2.10	2	0.33	0.28	0.39	2.07	2	0.28	0.36	0.37	2.09	2
Lu’an	0.28	0.59	0.13	1.84	2	0.38	0.32	0.30	1.93	2	0.35	0.46	0.19	1.84	2
Anqing	0.35	0.53	0.12	1.77	2	0.45	0.34	0.21	1.76	2	0.41	0.44	0.16	1.75	2
Ma’anshan	0.15	0.28	0.56	2.41	3	0.18	0.18	0.65	2.47	3	0.17	0.23	0.61	2.44	3
Wuhu	0.14	0.43	0.42	2.28	2	0.23	0.23	0.54	2.31	3	0.19	0.32	0.49	2.30	3
Xuancheng	0.46	0.45	0.09	1.63	2	0.65	0.22	0.14	1.49	1	0.56	0.32	0.11	1.55	1
Tongling	0.19	0.52	0.29	2.11	2	0.27	0.25	0.48	2.22	3	0.23	0.37	0.39	2.16	2
Chizhou	0.54	0.32	0.14	1.60	2	0.63	0.21	0.16	1.53	1	0.59	0.26	0.15	1.56	1
Huangshan	0.54	0.29	0.17	1.63	2	0.65	0.15	0.20	1.55	1	0.60	0.21	0.19	1.58	1

^1^ LE represents the level eigenvalue method, ^2^ AR represents the attribute recognition method.

**Table 4:** Average set pair potential based on subtraction of each index for evaluating water resources carrying capacity from 2011 to 2015 among different areas in Anhui Province.

Evaluation Index	Northern Anhui	Middle Anhui	Southern Anhui
*X*_1_ water resources per capita (m^3^/person)	−1.00	−0.21	0.02
*X*_2_ production modulus of water resources (10^4^ m^3^/km^2^)	−1.00	−0.64	0.12
*X*_3_ water supply per capita (m^3^/(person · year))	−0.49	0.85	0.69
*X*_4_ rate of vegetation coverage (%)	−0.98	−0.07	0.17
*X*_5_ daily domestic water consumption per capita (L/(person · day))	0.57	0.37	0.05
*X*_6_ water consumption per 10^4^ yuan (m^3^/10^4^ yuan)	0.83	0.42	0.57
*X*_7_ water consumption per 10^4^ yuan of value-added by industry (m^3^/10^4^ yuan)	0.26	0.31	−0.31
*X*_8_ density of population (person/km^2^)	−0.99	−0.43	0.06
*X*_9_ rate of urbanization (%)	0.78	0.67	0.55
*X*_10_ water consumption per mu for agricultural irrigation (m^3^/mu)	0.73	0.01	−0.45
*X*_11_ rate of water resources utilization (%)	−0.32	0.26	0.00
*X*_12_ gross domestic product per capita (10^4^ yuan/person)	−0.26	−0.01	0.75
*X*_13_ rate of urban wastewater treatment (%)	0.60	0.31	−0.21
*X*_14_ rate of water-saving irrigation area (%)	−0.58	−0.63	−1.00
*X*_15_ rate of ecological water consumption (%)	−0.95	−0.91	−0.41
